# Patients with geriatric syndromes and anti-amyloid therapies: lack of consideration? An exploratory analysis of the literature

**DOI:** 10.3389/fnagi.2023.1256968

**Published:** 2023-10-10

**Authors:** Angélique Sadlon, Martin Ott, Reto W. Kressig

**Affiliations:** ^1^Department of Clinical Chemistry, Inselspital, Bern University Hospital & University of Bern, Bern, Switzerland; ^2^Ageing Epidemiology Research Unit, School of Public Health, Imperial College London, London, United Kingdom; ^3^Memory Clinic Entlisberg, Geriatric Department City of Zurich, Zurich, Switzerland; ^4^University Department of Geriatric Medicine FELIX PLATTER & University of Basel, Basel, Switzerland

**Keywords:** anti-amyloid, geriatric syndrome, geriatric, Alzheimer’s disease, disease-modifying therapies, inclusion

## Abstract

**Introduction:**

Patients who should benefit from anti-amyloid therapies (AAT) are found across all geriatric settings. Yet, it remains unclear how the use of AAT in patients with geriatric syndromes, such as frailty and polypharmacy, has so far been discussed in the literature.

**Methods:**

Articles on aducanumab, gantenerumab, lecanemab, donanemab, crenezumab, solanezumab were retrieved in MEDLINE from inception to July 2023. For each article, identified geriatric relevant terms were assigned to five discussion contexts (eligibility of AAT study population, safety, prescription, patient clinical profile, alternative outcomes measurement). Article type and the involvement of geriatric healthcare professionals as an author were further extracted.

**Results:**

Out of 538 articles, 23 (4.27%) were published in journals from the geriatric category, 44 (8.18%) included an author affiliated with a geriatric institution. One hundred and sixteen (21.56%) articles included at least one geriatric relevant term, which were mostly discussed in the context of safety and eligibility. Articles mentioning geriatric syndromes were more frequently authored by a geriatric healthcare professional (*p* = 0.044).

**Discussion:**

The use of AAT in patients with geriatric syndromes has so far received poor attention in the literature raising concerns on their use in this patient group. The involvement of geriatric healthcare professionals in future studies may increase the relevance of AAT research in patients with geriatric syndromes.

## 1. Introduction

The discovery and development of monoclonal antibodies (mAb) targeting amyloid-β deposition, the pathological hallmarks of AD pathogenesis, has generated hope for a disease-modifying therapy in AD (Cummings et al., 2022). Supported by large media coverage, these anti-amyloid therapies (AAT) are concentrating increasing attention, and results from clinical trials are scrutinized with considerable interest. In 2021, the FDA approval of aducanumab, the first anti-amyloid mAb, for mild cognitive impairment (MCI) and early AD was met with enthusiasm but soon questions emerged regarding the clinical benefit (Knopman et al., 2021), the study populations (Cummings et al., 2021; Kuller and Lopez, 2021), the occurrence of side effects such as the amyloid related imaging abnormalities (ARIA) (Salloway et al., 2022), as well as the associated healthcare costs of initiating and monitoring such therapies (Whittington et al., 2022). While aducanumab authorisation by the European Medicines Agency (EMA) has been withdrawn in 2022 (EMA, 2022), new AAT have emerged: in early 2023, lecanemab gained FDA approval and it is expected that other AAT will seek regulatory approval in the coming year (Larkin, 2023). Individuals who may benefit from AAT are highly prevalent across all geriatric settings, from geriatric outpatient clinics to assisted living facilities (Bai et al., 2022; Chen et al., 2023). Geriatric syndromes, such as frailty and polypharmacy are very common in geriatric settings and are a known risk factor for adverse drug events (Zazzara et al., 2021). It remains unclear to what extent the use of AAT in patients with geriatric syndromes has so far been discussed in AAT literature. The aim of this study was to identify and characterize AAT articles mentioning geriatric relevant terms. We hypothesized that the use of AAT in patients with geriatric syndromes received poor attention so far in the literature.

## 2. Materials and methods

### 2.1. Anti-amyloid therapies and geriatric syndromes definitions

We considered the following mAb: aducanumab, gantenerumab, lecanemab, donanemab. Crenezumab, solanezumab. Geriatric relevant term (“geriatric term” in the text) included “geriatric” and any of the following geriatric syndromes: frailty, fall history, malnutrition and sarcopenia, urinary and faecal incontinence, depression, chronic pain, polypharmacy, polymorbidity, mobility/gait impairment, hearing and or vision impairment.

### 2.2. Search strategy

In a first step, articles were identified in MEDLINE from inception until 04.07.2023. Final search terms included “aducanumab [Title/Abstract] OR lecanemab [Title/Abstract] OR gantenerumab [Title/Abstract] OR donanemab [Title/Abstract] OR crenezumab [Title/Abstract] OR solanezumab [Title/Abstract].” We applied no restriction to article types and included those in English.

### 2.3. Data extraction

In a second step, for each retrieved article in MEDLINE, we searched for the presence of our terms of interest, using truncation, wildcards, and regular expression. Once a hit was identified we extracted the five words before and after that hit. These were then manually reviewed and checked for the occurrence of our terms of interest. Terms located within references and affiliation names where disregarded. In order to identify in which context the geriatric term were mentioned in the articles, we defined five discussion contexts: eligibility of AAT study population (ie the term is discussed in relation to AAT trials’ inclusion and exclusion criteria), safety (ie the term is discussed as an AAT side effect), prescription (i.e., the term is discussed in the context of AAT administration and monitoring), patient clinical profile (ie the term is mentioned as an AD clinical characteristic and risk factor), alternative outcomes measurement (i.e., the term is discussed as an alternative outcome to the ones measured in AAT trials). For each article, the occurrence of a term within any of the five contexts was counted once. To further characterize articles mentioning geriatric syndromes, we identified whether these articles were published in journals from the category “Geriatrics and Gerontology” (“geriatric category” in the text”) as defined by the Clarivate Web of Science classification. For each publication, we also ascertained whether any of the authors was affiliated with a geriatric institution. The latter was defined as containing any of the following words in the affiliation: “aging,” “ageing,” “geriatric,” “geronto,” “memory,” “old age,” “veteran.” Previous reports have suggested that article types are directly associated with the impact of an article (Chien et al., 2019). Therefore, we extracted the article types in which the terms were mentioned using the article type classification provided by MEDLINE: clinical trials, randomized clinical trials, review, editorial, news, meta-analysis, letter, case reports. All other articles were grouped into “other journal articles.”

### 2.4. Statistical analysis

In a third step, we compared the characteristics of articles mentioning geriatric relevant terms and articles not mentioning geriatric relevant terms. Differences between the two categories were evaluated using the Pearson’s Chi Square test. We applied a Mann-Kendall trend tend to assess the significance of change over time. Analyzes and visualization were undertaken in R version 4.2.2, using the packages ggplot2, circlize and Kendall. Results are presented as *N* (%).

## 3. Results

**Figure 1:**
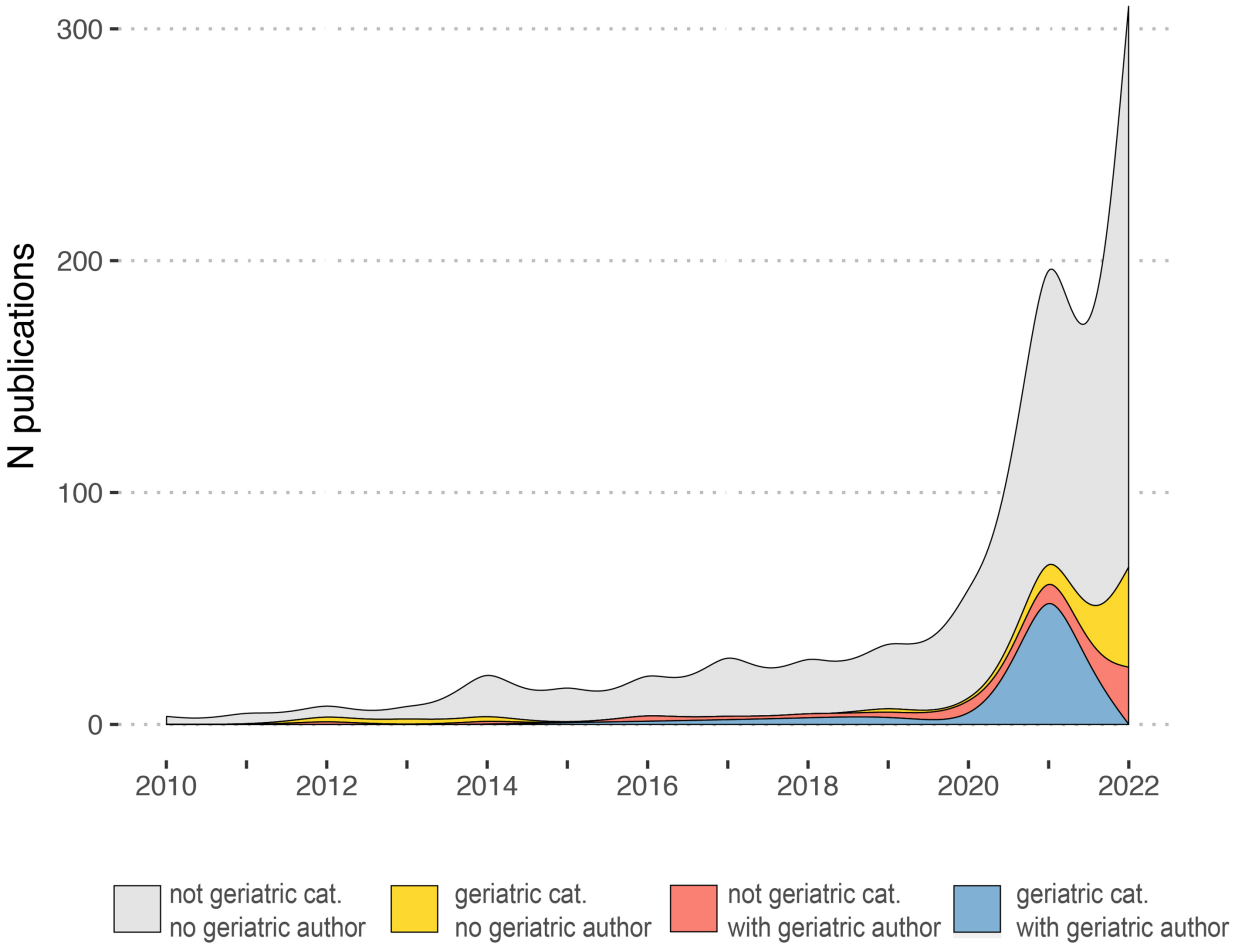
Number of publications on anti-amyloid therapies published in MEDLINE between 2010 and 2022. Different colors show whether a geriatric healthcare professional (“geriatric author”) was an author and whether the journal belonged to the category “Geriatrics and Gerontology” (“geriatric category” in the text) from the Clarivate Web of Science classification.

**Figure 2:**
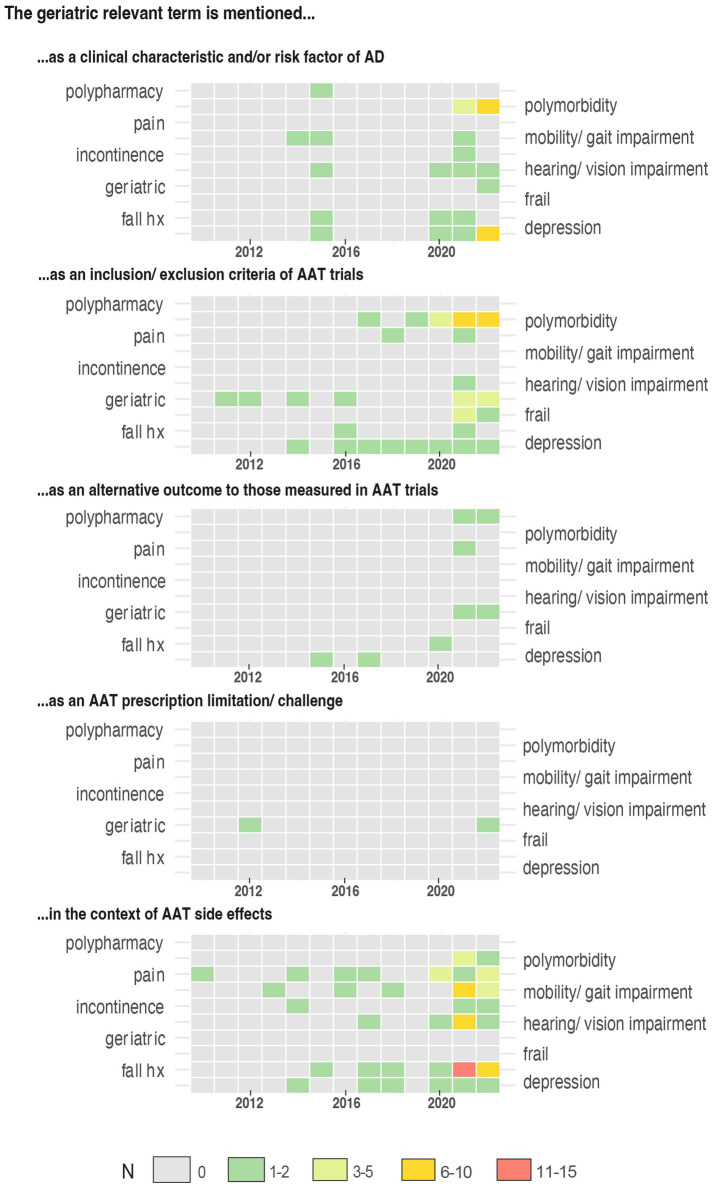
Timeline representing the number of papers published from 2010 to 2022 mentioning a geriatric relevant term, grouped according to five predefined discussion contexts.

**Figure 3:**
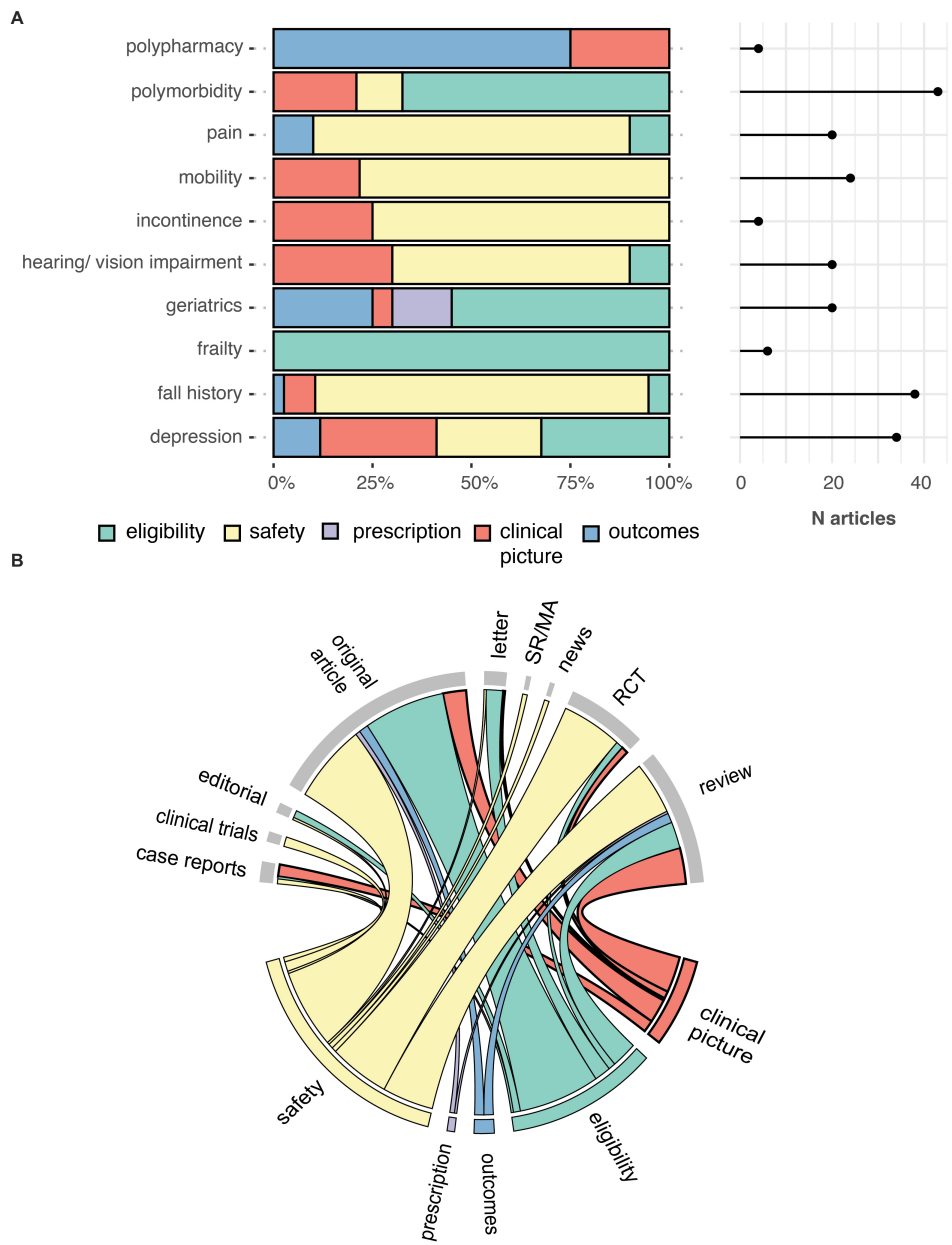
Number of times a geriatric relevant term was mentioned at least once in an article, by five contexts **(A)**. Chord diagram showing the link between the contexts in which geriatric relevant terms are mentioned (bottom) and the MEDLINE article type (top) **(B)**. Outcomes, alternative outcome measurement; RCT, randomized controlled trials; SR/MA, systematic review and meta-analysis.

Our search criteria identified 538 studies, among these, 23 (4.27%) were published in journals from the “Geriatrics and Gerontology” category and 44 (8.18%) articles included at least one author affiliated with a geriatric institution. The number of articles on AAT increased significantly over time for all articles (*p* < 0.0001), but not for articles published in the geriatric category (*p* = 0.057) (Figure 1). Similarly, an increase in articles on safety concerns and eligibility of AAT study population was found over time (Figure 2). From the 1,350 hit sentences containing previously defined geriatric terms, 417 (30.89%) were manually identified as being relevant to our search question (the remaining hits were either located within references, assessment names (geriatric depression scale) or within institution names) with 116 (21.56%) different articles mentioning at least one geriatric term. These articles were more frequently co-authored by geriatric medicine professional (*p* = 0.044) (Table 1). Polymorbidity (*n* = 43, 37.07%), followed by positive fall history (*n* = 38, 32.76%) were the most frequently mentioned geriatric syndromes (Figure 3). Neither the terms malnutrition nor sarcopenia were found in the retrieved articles. We observed that all terms, except polypharmacy, were mentioned at least once in the context of AAT safety and/ or eligibility (Figure 3). A subgroup analysis by article types found that clinical trials and randomized controlled trials were mainly discussing geriatric terms in the context of safety while letters discussed these mainly in the context of eligibility (Figure 3).

**Table 1:** Differences between articles mentioning geriatric terms and those not mentioning geriatric terms.

	Articles without geriatric relevant term mentioned *N* = 422^a^	Articles with geriatric relevant term mentioned *N* = 116^a^	*p* ^b^
*Drug*			0.5
Aducanumab	241 (57%)	71 (61%)	
Crenezumab	16 (3.8%)	7 (6.0%)	
Donanemab	22 (5.2%)	7 (6.0%)	
Gantenerumab	25 (5.9%)	4 (3.4%)	
Lecanemab	40 (9.5%)	12 (10%)	
Solanezumab	78 (18%)	15 (13%)	
*Clarivate journal category*			0.064
Geriatric and Gerontology	14 (3.3%)	9 (7.8%)	
Other	408 (97%)	107 (92%)	
*Author with geriatric institution affiliation*			0.044
No	400 (95%)	104 (90%)	
Yes	22 (5.2%)	12 (10%)	
*Article type*			0.0003
Case reports	2 (0.5%)	4 (3.4%)	
Clinical trials	9 (2.1%)	2 (1.7%)	
Editorial	31 (7.3%)	3 (2.6%)	
Letter	22 (5.2%)	4 (3.4%)	
Meta-analysis/ systematic review	4 (0.9%)	2 (1.7%)	
News	4 (0.9%)	1 (0.9%)	
Other journal article	225 (53%)	55 (47%)	
Randomized controlled trials	13 (3.1%)	15 (13%)	
Review	110 (26%)	30 (26%)	

a *n* (%).

b Fisher’s exact test; Pearson’s Chi-squared test.

## 4. Discussion

Accumulating evidence points towards an association between geriatric syndromes and the risk for adverse drug reactions (Zazzara et al., 2021). In this study we investigated how the literature on AAT has so far discussed the use of AAT in patients with geriatric syndromes. We identified that only about one in five articles on AAT mentioned at least once a geriatric term, suggesting that the use of AAT in patients with geriatric syndromes is receiving poor attention. Geriatric terms were most frequently discussed in the context of safety (i.e., as an occurring side effect of AAT) and eligibility (i.e., as an exclusion criteria in landmark clinical trials), two critical discussion points when using novel drugs in older adults (Lau et al., 2022). Fall (symptom of frailty) was among the most frequently mentioned geriatric term and was mainly discussed in the context of safety. This may be explained by the increased number of falls reported in patients receiving aducanumab (Sandrock, 2020) (15% vs. 11.8% in placebo) or lecanemab (van Dyck et al., 2023) (10.4% vs. 9.6% in placebo). Interestingly, frailty was reported in only six articles solely in discussions about the inclusion of frail patients in AAT trials. The fact that frailty is poorly addressed in the AAT literature is concerning as frailty is highly prevalent in MCI and dementia patients, and a known risk factor for adverse outcomes such as falls (Koria et al., 2022). Most importantly, current data from AAT trials do not allow to draw appropriate conclusion on their use in frail patients. As an example, one study conducted in an ambulatory geriatric unit found that only six out of 911 patients met the inclusion and exclusion criteria (including general health condition assessed using geriatric assessments tools and clinical frailty scales) from landmark trials on AAT (Canevelli et al., 2021).

Articles mentioning at least once a geriatric relevant term more frequently involved an author affiliated with a geriatric institution, suggesting that considerations on the use of AAT in patients with geriatric syndromes remain confined to the geriatric medicine field. In the last decade, collaborative research, involving experts with various backgrounds, has gained increased importance as a means to increase the diversity of participants and hence the generalisability of results (Coles et al., 2022). Failure to include key players in a field may hamper the inclusion of participants and alter the relevance and outcome of research. In our study, we identified the involvement of geriatric healthcare professionals through their affiliations. While we cannot exclude that some may have provided guidance at different stages of the articles without meeting the authorship requirements, a recent study suggests that authorship remains a good indicator of substantial involvement in the research being published (Reza et al., 2020). This study showed that in clinical trials and guidelines on heart failure, the presence of female authors was an independent predictor of the enrolment of female participants in studies on heart failure (Reza et al., 2020).

Noteworthy, less than 10% of the literature on AAT has been published in journals from the “Geriatrics and Gerontology” category. This may be explained by the novelty of the drug and lack of wide clinical implementation as well as the choice of authors to publish in journals with a wider audience. However, it may also translate the geriatric field’s concerns regarding current evidence on AAT as illustrated by recent statements from The American Geriatrics Society (AGS) or national stakeholders (Vellas, 2021; Harper and Lundebjerg, 2022; Kressig, 2022). This includes benefits-risk assessments of AAT as well as ethical and financial related considerations.

The scientific literature but also mainstream media have focused much attention on the occurrence of ARIA in patients under AAT (Hohman et al., 2016; Manly and Deters, 2023). These MRI abnormalities are characterized by oedema, effusion, haemosiderosis and/or microhaemorrhages (Hampel et al., 2023). Although mainly asymptomatic, ARIA can also induce neurological symptoms which may require a hospitalization. In rare cases, ARIA may also lead to death (Piller, 2023). Consequently, the FDA has recently issued a black box warning for lecanemab and the risk for ARIA (particularly in *APOE* ε4 homozygous carriers) and has prompted the need to appropriately weigh the risk-benefits of starting such therapy. The research of the pathophysiology behind ARIA is ongoing; so far, the presence of pre-existing microhaemorrhages and *APOE* ε4 carrier status has been identified as risk factors of ARIA (Hampel et al., 2023). While our study indicates that geriatric syndromes are most frequently discussed in the context of safety, to date, no study has investigated whether the presence of geriatric syndromes (such as frailty) could further increase the risk for ARIA. Though, a past study found that frailty is associated with cerebral microbleeds, a major risk factor for ARIA (Chung et al., 2016). Similarly, the role of polypharmacy, another geriatric syndrome, as a risk for ARIA remains unexplored. Past studies reported that anticoagulation use increases the risk for ARIA (Cummings et al., 2021, 2023). However, a cross sectional analysis of the Health and Retirement Study 2014 showed that at least one geriatric syndrome was found among 70% of individuals under anticoagulation use due to atrial fibrillation (Shah et al., 2021). Therefore, the discussion on the occurrence of ARIA should further extend to patients with geriatric syndromes.

Nevertheless, while concerns on the use of AAT in geriatric patients should not be ignored, they should not be an excuse for not discussing AAT in geriatric populations. Rather, they should highlight the importance of an interdisciplinary collaboration with all the actors in the field. In the last decade, the geriatric field has already gained some experience with the implementation of novel drugs in clinical practice, such as direct oral anticoagulants (Lee et al., 2012; Lip et al., 2021). To allow AAT to meet their expectations in geriatric settings, following steps should be considered: 1) The profile of individuals with adverse events in clinical trials should be clarified to identify whether preventive measures could be started before treatment initiation. For instance, in individuals experiencing falls under AAT, it may have been helpful to screen for drugs associated with increased risk for falls in older adults and replace them by a suitable alternative (de Jong et al., 2013). Furthermore, in patients eligible for AAT, geriatric assessment and gait analysis in fall clinics or geriatric outpatient clinics could help identifying other risk factors for falls beforehand and initiate preventive measures prior to the start of the AAT (Racey et al., 2021). 2) Promoting the inclusion of geriatric medicine healthcare professionals when AAT treatment is discussed in patients with geriatric syndromes. In these patients, a comprehensive geriatric assessment, may help identifying patients at high risk for adverse events, similarly to what is being implemented in oncology or cardiology (Pilotto et al., 2017). 3) Create an interdisciplinary network at local level which can coordinate the monitoring of AAT, with a special attention to geriatric settings, such as assisted living facilities.

Our study is associated with some limitations. First, we identified healthcare professionals from the geriatric field using institutional affiliations name, it is possible that geriatricians work outside of a geriatric institution. It is also possible that geriatricians were involved in the conception of an article and provided insights, without fulfilling the requirements of an authorship. Nonetheless, we believe that authorship reflects a substantial involvement in the research, which may have resulted in geriatric relevant considerations being more likely to be addressed. Second, we focused on articles written in English, it is possible that we missed articles written in other languages. Third, our search was limited to MEDLINE and did not include articles from the gray literature. We note however, that our study’s aim was to provide a topical rapid review of the literature on an ongoing debate regarding the use of AAT. A previous meta-epidemiological study showed that for such research questions, MEDLINE can be considered as a comprehensive data source (Marshall et al., 2019). Finally, our study’s findings could be strengthened by the analysis of individual patient data from large clinical trials or adverse event reports [e.g., from the FDA Adverse Event Reporting Systems (FAERS)]. Currently, not all data is publicly available and adverse events reports on FAERS remains too low to draw appropriate conclusions.

## 5. Conclusion

In conclusion, this study provides important insights on the attention geriatric syndromes have so far received in the AAT literature. In the coming years, AAT and possibly other disease-modifying therapies may become game changers in the treatment of dementia, which may inflict a significant challenge to our healthcare system. Only a strong interdisciplinary collaboration including experts from the geriatric field will help AAT and other disease-modifying therapies keep their promises.

## Data availability statement

The raw data supporting the conclusions of this article will be made available by the authors, without undue reservation.

## Author contributions

AS: Conceptualization, Data curation, Formal analysis, Investigation, Methodology, Visualization, Writing – original draft, Writing – review & editing. MO: Conceptualization, Data curation, Writing – review & editing. RK: Conceptualization, Supervision, Writing – review & editing.
